# Gifted and Average-Ability Children’s Potential for Solving Analogy Items

**DOI:** 10.3390/jintelligence7030019

**Published:** 2019-08-27

**Authors:** Bart Vogelaar, Sophie W. Sweijen, Wilma C. M. Resing

**Affiliations:** Developmental and Educational Psychology, Leiden University, 2300 RB Leiden, The Netherlands

**Keywords:** analogical reasoning, dynamic testing, gifted, graduated prompts

## Abstract

Analogical reasoning is assumed to play a large role in learning and problem solving in everyday and school settings. It was examined whether a newly developed dynamic test of analogical reasoning would be sufficiently difficult for identifying young gifted children’s potential for solving analogies. The study included 74 gifted (*n* = 31) and average-ability (*n* = 43) children between 7 and 8 years old. Employing a pre-test–training–post-test format, in which half of the children received a graduated prompts training and the other half received a control task between pre-test and post-test, it was investigated (1) whether trained children would improve more in accuracy from pre-test to post-test than their untrained peers, and whether (2) gifted and average-ability children would demonstrate differences in their level of improvement from pre-test to post-test, and (3) their needs for instruction during training. The results indicated that dynamically tested children improved more than those in the control condition. In addition, the dynamic test seemed sufficiently difficult for the gifted children: regardless of whether they were trained, gifted children demonstrated superior accuracy scores than their average-ability agemates at pre-test and post-test, but similar levels of improvement. They were also found to need fewer instructions during training.

## 1. Introduction

Analogical reasoning refers to a cognitive process in which information from a known source is identified and transferred to a new information system [1,2]. It is considered a core component of learning and problem solving in everyday situations [2]. More specifically, researchers have argued that this form of reasoning is related to a variety of cognitive skills and processes [3,4], including fluid intelligence [5,6]. In general, performance on analogical reasoning tasks has been found to be influenced by three types of factors: the structure of the items, such as the similarity of the different elements of an item, characteristics of the problem solver, and specific task factors, for instance related to context and cognitive processing load [7]. With regard to the characteristics of the problem solver, often cited factors include expertise and general ability. Indeed, various research has shown that children of high ability outperform those with lower intelligence scores in their performance on analogical reasoning tests [8,9,10].

An important consideration in analogical reasoning research concerns the manner in which performance is measured. Often, single-occasion, also called static, testing is used to assess how well children can reason by analogy, for example as part of a traditional intelligence test [11]. However, these tests fail to take into account that children demonstrate great intra-individual cognitive variability in their mastery of a certain skill [12]. Moreover, these tests have been noted for underestimating the ability of certain children, for example those who have a different ethnic or linguistic background or come from low socioeconomic environments [13]. An alternative means of assessing children’s ability is dynamic testing, which is an interactive approach to psycho-educational assessment that integrates feedback or help into the testing procedure [13,14]. Many dynamic tests include a specific training session in which this feedback and help is provided, and often follow a test–training–test format [14]. By focusing on how much a child can learn, rather than on how much a child has already learned, these tests are said to provide insight into children’s potential for learning [15].

Research into the dynamic testing of gifted children emerged in the 1980s as a response to the observation that gifted identification based on static testing might result in an underrepresentation of children from low socioeconomic [16] or diverse linguistic or cultural backgrounds [17]. Traditionally, it was often thought that these children could manage their learning on their own [18]. Various studies have, however, suggested that children with high ability can also profit from a dynamic approach in demonstrating their potential [19,20,21]. In general, research has supported the view that in comparison with their typically developing peers, gifted children’s learning can be characterized by higher performance before and after training, more improvement from pre-test to post-test, as well as an advantage in transfer [22,23]. However, measuring gifted children’s potential for learning has proved challenging due to the occurrence of ceiling effects [22,23,24]. Therefore, the current study aimed to investigate whether a newly developed dynamic test of analogical reasoning contained items of sufficient complexity to identify the potential for reasoning by analogy of young gifted children. In order to investigate whether gifted children would outperform their typically developing peers in their potential to solve analogies, their performance was compared with a group of average-ability children.

### 1.1. Dynamic Testing of Analogical Reasoning

The theoretical background of dynamic testing can, for a large part, be traced to the work of Vygotsky [14,25], who posited that children’s learning occurs in a zone of proximal development (ZPD). As part of the concept of the ZPD, learning can be seen as a social construct, with children learning with and from ‘expert’, more capable others. The ZPD has been defined as, on the one hand, the actual level of development, which refers to independent learning or problem solving, and, on the other, the potential level of development, referring to the level of cognitive functioning a child reaches with help from the more capable other person. The actual level of development of a child, as measured by a static test, is not always indicative of their potential, whereas a dynamic test with a pre-test–training–post-test format is said to provide insight into both a child’s actual level of development (the pre-test) and the potential level of development (the post-test), thereby tapping into children’s potential for learning [13].

The second aim of dynamic testing is providing insight into children’s individual instructional needs arising during training [26]. These instructional needs are said to be indicative of a child’s educational needs in the classroom [27]. An approach that has been used successfully for these purposes is the graduated prompts intervention [28,29]. This approach is different from other training approaches used in dynamic tests, such as those devised by Feuerstein [14], in that it is highly standardized but, at the same time, can be individualized based on the perceived needs of the testee. Graduated prompts training consists of providing a hierarchically ordered series of prompts as soon as a child demonstrates difficulty in independent problem solving. Each time a child makes a mistake in problem solving, he or she is provided with a prompt that becomes increasingly more specific, ranging from metacognitive, general prompts, to more specific cognitive prompts, with the final prompt consisting of step-by-step modelling [30]. Metacognitive prompts often provide children with self-regulatory strategies, for example by activating their prior knowledge (e.g., ‘how did you solve these tasks last time?’). Cognitive prompts are tailored to individual items and teach children aspects of the task-solving process (e.g., ‘what are the similarities between these two elements of the task?’). In doing so, this procedure is believed to provide insight into the different degrees of help individual children need in order to show progression in learning [26].

**Figure 1:**
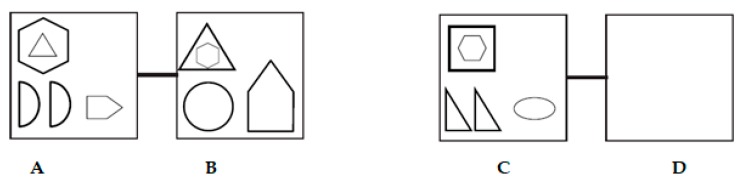
Example of a difficult analogy item.

Graduated prompts protocols are often based on a task analysis of the test items. In dynamic tests of analogical reasoning, the more specific prompts provided during training are related to the processes of analogical reasoning. The current study utilized a dynamic test employing visual–spatial geometric analogies of the type A:B::C:D. A sample item can be found in Figure 1.

In the graduated prompts procedure employed in the current study, based on Sternberg’s process model of analogical reasoning [6], children are first prompted to encode the A and B terms of the analogy, which, in Figure 1, refer to the first two boxes, and use inferences to find the commonalities and differences (transformations) between A and B. In Figure 1, six transformations are visible. The hexagon and triangle in A become larger (two transformations), the two half circles in A become one full circle in B (two transformations: from half to whole and from two to one element), and the pentagon rotates and becomes larger (two transformations). Then, they are prompted to encode the C term, the third box in Figure 1, after which they are prompted to map the commonalities and differences between A and B, and apply these to C. Then, they are encouraged to encode the D term of the analogy and provide the correct solution in the fourth box (D) [6,31]. Mapping is considered one of the core processes of analogical reasoning and involves finding out the commonalities between two situations that make up the analogy: the original situation (the ‘base’) and the new situation (the ‘target’) [1,7,31]. The next stage of mapping consists of information that needs to be inferred from the base to the target. In order to do so, the base and the target need to be ‘aligned’ on the basis of their similarities, and inferences will be made on the basis of that alignment. In Figure 1, A would need to be aligned with C and B with D.

Various studies have investigated dynamic tests employing analogy items of the type A:B::C:D. In such studies, it was found that dynamic measures of analogical reasoning, consisting in these studies of the post-test accuracy score as well as the total number of instructions young children needed during training, predicted math and reading performance [32,33]. Moreover, children’s improvements in performance from pre-test to post-test have been found to be unique predictors of scholastic performance, predicting children’s performance above and beyond static measures of analogical reasoning [14,26].

With regard to the use of dynamic tests of analogical reasoning for gifted children, the research is sparse. Results of studies amongst 5 to 8-year-old gifted and average-ability children demonstrated that gifted children, in general, outperformed their average-ability peers in the extent to which they solved analogy items correctly [24] or could accurately apply analogical transformations [34]. These two groups of children, however, showed similar levels of improvement after a graduated prompts training, which was unexpected in the light of dynamic test outcomes in the domains of complex problem solving [22], visual–spatial memory and perceptual structuring [19]. According to the authors, the similar level of improvement of the two groups of children could partially be ascribed to a potential ceiling effect amongst (some of) the gifted children [24,34]. The authors of these studies also investigated children’s need for instruction during a graduated prompts training, and reported that, at the group level, the two groups of children needed an equal number of instructions. In both ability groups, however, there were large individual differences in the number of instructions the children required. In a different study, 10 and 11-year-old children’s potential for learning was measured by means of a dynamic test of visual analogies [34]. In this study, children were categorized into four groups based on their school performance and general tests of cognitive ability: gifted children, children with exceptionally high and exceptionally low performance, and typically performing children. The authors found that at both the pre-test and post-test, gifted children achieved significantly higher accuracy scores than the children who were classified as exceptionally low performing. In relation to the use of strategies, it was reported that both gifted and exceptionally high performing children demonstrated a significantly higher use of strategies at the pre-test and the post-test than their peers in the other groups. As group differences in accuracy scores and use of strategies were less pronounced after training than before, the authors concluded that dynamic testing has the potential to minimize differences in initial performance, which they termed the equalizing effect of dynamic testing.

All in all, these three studies demonstrate that children of different ability levels, even though some may already perform at advanced levels, can in fact improve their analogical reasoning ability, if they are provided with the right instructions. With regard to testing gifted children dynamically, utilizing test items with sufficient complexity seems to be of the utmost importance to obtain insight into their potential for solving analogies [22,24,34].

### 1.2. The Current Study

In previous studies in which dynamic tests of analogical reasoning were used to investigate the potential for learning of gifted children, it was suspected that the test items used may not have been of sufficient difficulty, possibly resulting in a ceiling effect [22,23,24]. Therefore, we developed a new dynamic test of analogical reasoning, utilizing more complex items. The current study investigated whether our newly developed dynamic test of analogical reasoning would be of sufficient difficulty so that it could be used in unveiling the potential for solving analogies of young gifted children. Our first task was to analyze the effectiveness of the newly developed dynamic test in improving children’s ability to reason by analogy in comparison with static testing (control group, who completed the pre-test and post-test only) for the two ability groups (gifted and average-ability). It was expected that children who were dynamically tested would show more improvement in the number of accurately solved analogy items from pre-test to post-test than their untrained peers in the control group [24,26,29,30,34]. As to potential differences between gifted and average-ability children, it was expected that gifted children would show more improvement after training or control [19,20], as well as higher scores at pre-test and post-test [24,34].

Secondly, we focused on children’s need for instructions during the graduated prompts training. Firstly, potential changes in their instructional needs from the first to the second training session were analyzed. It was expected that all children would demonstrate a decrease in their need for instructions from the first to the second training session [30]. Moreover, it was expected that the gifted children would show a similar level of decrease, and an equal number of instructions as their average-ability peers [24,34].

## 2. Materials and Methods

### 2.1. Participants

The current study employed 74 children between seven and eight years old (*M* = 7.25, *SD* = 0.46). All children attended one of six primary schools in the western part of the Netherlands and participated on a voluntary basis. Children were divided in six different classes, one per school. Of all the children approached, 70% obtained permission to participate. All of them spoke Dutch as their first language and attended schools in middle or high socio-economic status neighborhoods. Their parents and the school’s headmasters had provided written informed consent prior to their participation in the study. Prior to the onset, the study was reviewed and approved by the university’s board of ethics. Three children were excluded from the data analysis because they did not complete all sessions. All children were assigned to one of the two ability groups: a group of gifted children (*n* = 31) and a group of average-ability children (*n* = 43).

#### Giftedness Identification Procedure

In the Netherlands, full scale intelligence testing is not commonly conducted in primary education. Therefore, in the current study, in accordance with the position statement of the National Association for Gifted Children (NAGC) [35], and Gagné’s Differentiated Model of Giftedness and Talent (DMGT) [36,37], children were categorized as gifted if they scored at least at the 90th percentile of the Raven Standard Progressive Matrices (RSPM) [38], administered as part of the current study. In order to make sure that there would be enough gifted participants, gifted children were oversampled and recruited from schools offering special educational provisions for gifted and talented children. All participating children who scored below the 90th percentile of the RSPM were categorized as average-ability, and those who scored at or above the 90th percentile as gifted.

### 2.2. Design

The study used a two-session (pre-test, post-test) repeated measures randomized blocking design with two experimental conditions: dynamic testing versus control. Table 1 displays an overview of the experimental design.

The RSPM [38] was administered before the dynamic/static test of analogical reasoning to ensure that possible differences in the initial reasoning abilities of the children were kept at a minimum. Pairs of children with equal Raven scores (blocks) per ability group, school and gender were randomly assigned to the two experimental conditions. This resulted in the following four subgroups: gifted dynamic testing (*n* = 14), gifted control (*n* = 17), average-ability dynamic testing (*n* = 22), and average-ability control (*n* = 21).

A graduated prompts training was administered to the children in the dynamic testing condition, between the pre-test and the post-test. This training consisted of two sessions of 20–30 min each. The children in the control condition were not provided with (training in solving) analogies during the training time-frame, but performed unrelated control tasks consisting of mazes and dots-to-dots completion tasks taking approximately the same amount of time to ensure a similar time-on-task exposure for the children in the two experimental conditions.

### 2.3. Materials

#### 2.3.1. Raven Standard Progressive Matrices

The RSPM [38] was used as a blocking instrument before the administration of the executive function measures and the dynamic test of analogical reasoning. The Raven is a test of inductive reasoning abilities using visual analogy items. High internal consistency is reported in the literature (split-half coefficient of *r* = 0.91) [38]. High internal consistency was also found in our sample of participants (α = 0.90).

#### 2.3.2. Dynamic Test of Analogical Reasoning

The newly developed dynamic test, consisting of a pre-test–training–post-test format, used consisted of visual–spatial geometric analogy items of the type A:B::C:?. These items were originally developed by Hosenfeld, Van den Boom, and Resing [39]. For the current study, these items were made more complex by adding new shapes (elements) and changes in the relations between the different elements (transformations). Each item consisted of a maximum of six different geometric shapes: ellipses, circles, triangles, squares, pentagons and hexagons. Each analogy item contained between two and fourteen different transformations, including the following transformations: adding or subtracting an element, changing position, changing size, halving, changing color, and rotation. Item difficulty was defined by the number of different elements as well as the number of transformations the elements undergo [40,41]. Children were instructed to draw their answers. See Figure 1 above for a sample item.

Both the pre-test and post-test consisted of 20 analogy items of varying difficulty. The pre-test and post-test were created as parallel versions, having equivalent items regarding the difficulty levels of the items and the order in which the items were presented. Children were instructed to solve the items independently without receiving feedback on their answers.

The graduated prompts training consisted of two short training sessions of six new analogies each, containing between two and five different elements and four to eight transformations. Using a graduated prompts technique as described in earlier studies [24,30], the prompts children received were provided hierarchically: two general metacognitive prompts, followed by three cognitive prompts tailored to the solution of each individual item. In case the child could not answer the item correctly after these prompts, the final prompt consisted of step-by-step guidance to the correct answer (modelling). After solving an item correctly or after having been provided with the final prompt, the children were asked to explain why they thought their answer was correct. Usage of these prompts was based on task analysis of the analogy solving process [6,32]. A schematic overview of the prompts can be found in Appendix A. The experimenters were trained extensively by the first author prior to administering the training, and interacted with the children following a strict protocol, to safeguard standardization of the training procedure. The experimenters did not know which of the ability groups the child was assigned to.

### 2.4. Procedure

Children were tested weekly in five different sessions. In the first session, the RSPM was administered. The second session consisted of the pre-test, the third and fourth session of training or the control task, respectively, and the fifth session of the post-test. All test sessions were administered individually within a protocolled procedure. Prior to the pre-test and post-test, children were asked to first name all the shapes used in the analogy items and then to copy the shape underneath the original one, under the assumption that this would activate prior knowledge and would ensure that the examiners and children used the same terminology. This procedure was believed to facilitate the scoring of children’s answers [42].

## 3. Results

### 3.1. Test Characteristics

First of all, we analyzed the psychometric properties of the dynamic test. For both the pre-test and post-test, for each individual item, the proportions of participants that solved the items accurately (*p*-values) were calculated, with values ranging from 0.06 to 0.67. Item-total correlations were revealed to be moderate to high, ranging from *r_it_* = 0.42 to *r_it_* = 0.77 for the pre-test, from *r_it_* = 0.45 to *r_it_* = 0.94 for the post-test of the children in the dynamic testing condition, and from *r_it_* = 0.52 to *r_it_* = 0.88 for those in the control condition. A complete overview of the p-values and item-total correlations can be found in Appendix B. Moreover, the internal consistencies of the pre-test and post-test were found to be high: α = 0.93 for the pre-test, and α = 0.95 and α = 0.96 for the post-test (control and dynamic testing condition, respectively). In addition, a higher test–retest correlation was found for the children in the control condition (*r* = 0.84, *p* < 0.001) than for those who were dynamically tested (*r* = 0.53, *p* < 0.05). Fisher’s *r* to *z* transformation indicated that the two correlation coefficients differed significantly, *z* = 2.62, *p <* 0.01.

### 3.2. Initial Group Comparisons

A multivariate analysis of variance (MANOVA) was conducted to detect possible differences between the two ability groups and the two experimental conditions in their age, Raven and pre-test scores. Multivariate results indicated that there were no significant differences in these variables between the children in the two conditions (Wilks’ λ = 0.99, *F* (3, 68) = 0.16, *p* = 0.921, η_p_^2^ = 0.01). Univariate results further demonstrated that no significant differences were found between the children in the dynamic testing and control condition in age (*p* = 0.759, η_p_^2^ = 0.001), Raven score (*p* = 0.510, η_p_^2^ = 0.01), and pre-test accuracy (*p* = 0.872, η_p_^2^ < 0.001).

With regard to potential differences between the two ability groups, multivariate results indicated significant differences (Wilks’ λ = 0.48, *F* (3, 68) = 24.27, *p* < 0.001, η_p_^2^ = 0.52). Univariate results further revealed that gifted and average-ability children did not differ in their age (*p* = 0.445, η_p_^2^ = 0.01), and pre-test accuracy (*p* = 0.07, η_p_^2^ = 0.05). However, gifted children outperformed their average-ability peers in Raven scores (*p* < 0.001, η_p_^2^ = 0.45), as was expected. The assumptions for conducting a one-way MANOVA were met. The variables demonstrated relative normality, and no significant outliers were identified. Moreover, Box’s test of equality of covariance matrices indicated there was homogeneity of variance (*p* = 0.57). Descriptive statistics of all measures, including information on minimum and maximum scores, kurtosis and skew are shown in Appendix B, divided by experimental condition and ability group.

### 3.3. Changes in Accuracy of Analogical Reasoning

A repeated measure analysis of variance (ANOVA) was performed to examine the effects of dynamic testing versus control only on children’s improvement in accuracy of analogical reasoning. Ability group (gifted/average-ability) and Condition (dynamic testing/control) were added as between-subject factors and Time (pre-test/post-test) as a within-subjects factor. The significant main effect of Time revealed that the accuracy scores of all groups of children improved significantly from pre-test to post-test (Wilks’ λ = 0.51, *F* (1, 70) = 67.85, *p* < 0.001, η_p_^2^ = 0.49, observed power (1 − β) = 1.00). In addition, children from the dynamic testing condition progressed significantly more in accuracy than children in the control condition, as indicated by the significant Time x Condition effect (Wilks’ λ = 0.85, *F* (1, 70) = 12.69, *p* = 0.001, η_p_^2^ = 0.15, observed power (1 − β) = 0.94), as well as a visual check of the mean scores.

**Figure 2:**
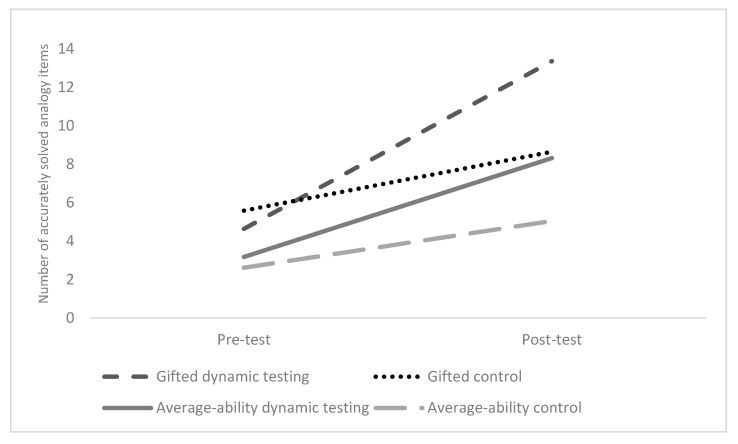
Improvement in accuracy in analogical reasoning at pre-test and post-test for each ability group and experimental condition.

However, as expected, we found no significant differences between gifted and average-ability children in their improvement from pre-test to post-test as witnessed by the non-significant Time × Ability group effect (Wilks’ λ = 0.96, *F* (1, 70) = 3.21, *p* = 0.077, η_p_^2^ = 0.04, observed power (1 − β) = 0.42). Moreover, the non-significant Time × Ability group × Condition effect suggested that there were no significant differences between the two ability groups in the effects of dynamic testing or static testing (control group) in relation to changes in accuracy from pre-test to post-test (Wilks’ λ = 0.98, *F* (1, 70) = 1.58, *p* = 0.213, η_p_^2^ < 0.02 observed power (1 − β) = 0.24). The between-subjects effect of Ability group, however, indicated, in accordance with our hypothesis, that gifted children had significantly higher accuracy scores at pre-test and post-test (*F* (1, 70) = 6.48, *p* = 0.013, η_p_^2^ = 0.09, observed power (1 − β) = 0.71). The progression lines in accuracy of analogical reasoning for both ability groups and experimental conditions are displayed in Figure 2.

### 3.4. Changes in Children’s Instructional Needs

To examine possible differences in the instructional needs of the children during both training sessions, we performed three separate repeated measures ANOVAs for metacognitive, cognitive, and modelling prompts, respectively, with Time (training 1/training 2) as within-subjects factors and Ability group (gifted/average-ability) as a between-subjects factor. These analyses allowed us to investigate potential differences between the two ability groups in the number of instructions they needed during the graduated prompts training, differentiating between the number of metacognitive, cognitive and modelling prompts they were provided with. The results of these analyses are shown in Table 2. A Bonferroni correction was applied to the α level to prevent Type I errors, resulting in a threshold of *p* < 0.016 necessary to reach significance.

None of the main effects of Time or the interaction effects of Time × Ability group was significant, which suggested, in contrast with our expectations, that neither gifted nor average-ability children showed a statistically significant decrease from the first to the second training session in the number of prompts they needed. The effect sizes of the main effects of Time for the number of metacognitive and cognitive prompts, however, were moderate.

**Figure 3:**
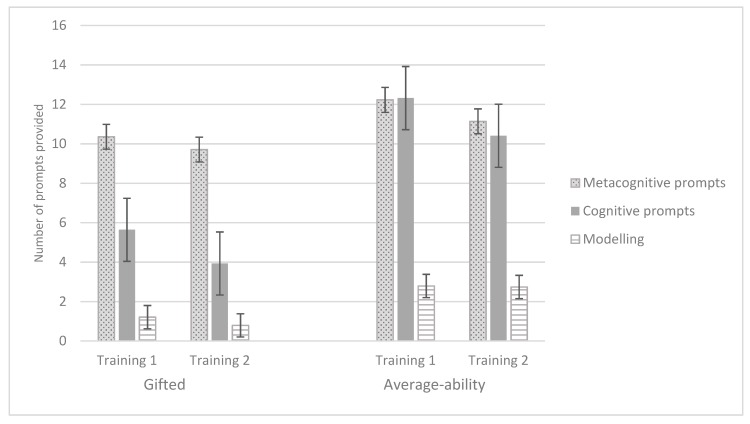
Number of metacognitive, cognitive and modelling prompts for both training sessions and ability groups. Error bars denote standard errors.

In addition, the between-subjects effects of Ability group for metacognitive prompts (*F* (1, 34) = 6.31, *p* = 0.017, η_p_^2^ = 0.16, observed power (1 − β) = 0.68) and modelling (*F* (1, 31) = 3.95, *p* = 0.056, η_p_^2^ = 0.11, observed power (1 − β) = 0.49) did not reach significance. However, the between-subjects effect of Ability group for cognitive prompts was significant (*F* (1, 34) = 7.93, *p* = 0.008, η_p_^2^ = 0.19, observed power (1 − β) = 0.78). The results indicated that gifted children needed fewer cognitive prompts than their average-ability peers, but, on average, the same number of metacognitive or modelling prompts. Mean scores and standard errors of children’s need for instructions can be found in Figure 3.

## 4. Discussion

As previous research has demonstrated that dynamic tests of analogical reasoning were not sufficiently complex for gifted children [24,34], the present study aimed to investigate whether a newly developed dynamic test of analogical reasoning could be used to measure the potential for learning of young gifted children. In doing so, we examined the psychometric properties of the test to assess whether the test was of sufficient difficulty for this group of learners. We focused on potential differences in the level of improvement in accurately solved analogies of gifted and average-ability children and potential differences in their needs for instruction during a graduated prompts training procedure.

In general, it was found that children who were provided with training improved more in solving analogies than their peers who completed the pre-test and post-test only. In combination with differences in the test–retest reliability analysis, these results suggest that our dynamic test might be successful in tapping into children’s potential for learning, as was also found in other studies utilizing an experimental design with two conditions, a training condition and a control condition provided with unrelated control tasks [24,30,33,34]. As part of the design of these studies as well as that of the current study, however, the children in the dynamic testing condition had more exposure to solving analogies than those in the control condition. As a result, we cannot rule out that the larger improvement of the children in the training condition was, in part, the result of practice effects. Nevertheless, in previous studies that included three experimental conditions—a training condition, a control condition that did not receive training but practiced with the same analogies as the children who were provided with training, and a control condition provided with unrelated control tasks during the training time-frames—it was revealed that the children who were provided with a graduated prompts training showed improvements beyond those who practiced only and those who completed control tasks [32,43]. In order to validate the usefulness of our graduated prompts procedure beyond that of mere exposure to solving analogies, future studies into our newly dynamic test should aim at utilizing four experimental conditions: two experimental conditions receiving training, one of which was a graduated prompts approach, a control condition practicing with the same analogies as the children in the training conditions, and a control condition provided with unrelated control tasks.

In addition, the results of the current study revealed that individual differences were found in the level of improvement after training. These results mirror findings of earlier studies utilizing dynamic tests of analogical reasoning and other subtypes of inductive reasoning [27,30,34]. The results of the current study further underline, more importantly, that individual differences were also apparent within the group of gifted children. This group of children demonstrated large inter-individual differences in performance before and after training as well as in levels of improvement, suggesting that gifted children are not, as was previously often assumed, a homogeneous group when it comes to their cognitive abilities [44]. Rather, they seem to have individual differences in their potential for learning, differences which can be unveiled by a dynamic test. Future studies could, therefore, investigate the nature of these individual differences in more detail, utilizing our newly developed dynamic test.

In this light, moreover, our dynamic test was also found to be sufficiently complex for gifted children, as supported by an analysis of children’s improvement in accuracy as a result of training and the p-values of the individual test items. An analysis of the mean scores, standard deviations and p-values suggested, however, that a ceiling effect did not occur in the current study. Gifted children outperformed their average-ability peers in accurately solved analogies, as was expected, but demonstrated similar levels of improvement. Although in previous studies similar levels of improvement at the group level were, in part, ascribed to a ceiling effect [24,34], the current study findings reveal that when no ceiling effect is present—the two groups of children still demonstrate a similar level of improvement, supporting these previous findings.

Large individual differences were also found in children’s instructional needs, as witnessed by the standard errors for the number of metacognitive, cognitive and modelling prompts portrayed in Figure 3. At the group level, however, significant differences were only found for the number of cognitive prompts. Gifted children needed fewer cognitive prompts than their average-ability peers, but a similar number of metacognitive and modelling prompts. In contrast with our expectations, the reduction in the number of prompts from training 1 to training 2 did not reach statistical significance, suggesting that the analogy items we used in the current study were sufficiently difficult for the (gifted) children. In earlier studies, in which a ceiling effect was deemed likely, gifted and average-ability groups needed an equivalent number of instructions during training [24,34]. Perhaps the relative complexity of the items in the current study posed a challenge for them, which, according to research in scholastic domains, would lead to gifted children being more motivated, and exhibiting persistence, elaborative processing, creativity and the willingness to take risks [45,46]. In turn, this process is assumed to foster self-regulated learning [47]. Of course, this tentative hypothesis requires more research, for instance in relation to the role motivation plays in improvements from pre-test to post-test and the instructions children need in a dynamic test setting.

In addition to those mentioned above, the current study encountered some additional limitations. As mentioned above, the number of participants per subgroup was low, which not only had an effect on the power of the analyses conducted, but also affects the generalizability of the results. In the Netherlands, intelligence testing often occurs only in special circumstances. Therefore, unfortunately, the current study employed a relatively small number of gifted children. An analysis of the effect sizes and observed power suggested that our study had enough power to detect differences between the two experimental conditions, but that the analyses regarding differences between ability groups and conditions may have been underpowered. Future research should therefore aim at employing a larger number of participants. Considering it is challenging to find large numbers of young gifted children, increasing the number of average-ability children would most likely also increase power [48]. Furthermore, it must be noted that, in accordance with the NAGC [35] and Gagnés DMGT [36,37], a cut-off score of the 90th percentile on the RSPM was used in the current study to categorize children as gifted. However, some of the average-ability children scored relatively close to the 90th percentile, making the difference between some of the average-ability and gifted children rather small. In future studies, therefore, a larger difference could be established between the cognitive abilities of participating gifted and non-gifted children. In addition, in the current study children were randomly assigned to two experimental conditions based on their initial inductive reasoning scores. Although randomization is seen as the only satisfactory method to control for the regression effect [49], it cannot be discounted that a regression effect still occurred in the current study, which could have affected the post-test scores of, in particular, the gifted children regressing to the mean. Future studies could include at least two pre-tests to control for regression to the mean occurring [49]. Such studies could, furthermore, focus on a larger age range to investigate the suitability of the newly developed test for children of different ages. Moreover, it would be interesting to identify high ability on the basis of dynamic measures of analogical reasoning, rather than on nominations and static measures of analogical reasoning. The next step in studies utilizing the same materials would be computerizing the dynamic test, which would allow for more fine-grained analysis of children’s processes in solving analogy items, and individualized adaptive testing.

In conclusion, our findings that the gifted children show individual differences in their level of initial performance, and subsequent improvement, in combination with differences in the amount and type of instruction they benefit from, indicate that these children do not form a homogeneous group, but have individual cognitive abilities and potential for growth. They might have the potential to excel but need instruction tailored to their needs to be able to unfold their potential. These conclusions underline the developmental nature of giftedness and support the notion that gifted children have an exceptional potential for learning new skills, if learning takes place within their individualized zone of proximal development [50]. These findings also bear implications for educational practice. Special educational provisions for high-ability students should have opportunities for differentiation at the level of the individual learner, both in terms of difficulty level of the content of the curriculum as well as in the instructions provided in class. Children’s initial ability is not necessarily predictive of their ability to learn, and the feedback they need from their teachers. The outcomes of the current study show that the abilities of children, irrespective of whether they are considered high or average-ability children, are malleable. Dynamic testing outcomes could be used as a means for teachers to think more flexibly about the abilities of their students. Dynamic tests can be used to identify individual learning paths and instructional needs and serve as a starting point for differentiating in the (difficulty level of the) content of the curriculum, for example in relation to suitable enrichment activities.

## Figures and Tables

**Table 1:** Overview of the design.

Condition	Groups	Dynamic/Static Test				
		Raven	Pre-Test	Training 1	Training 2	Post-Test
Dynamic testing	Gifted (*n* = 14)	X	X	X	X	X
	Average ability (*n* = 22)	X	X	X	X	X
Control	Gifted (*n* = 17)	X	X	Control task	Control task	X
	Average ability (*n* = 21)	X	X	Control task	Control task	X

**Table 2:** Results of the repeated measures ANOVA for changes in children’s instructional needs from the first to the second training session.

	Wilks’ λ	*F*	*p*	η_p_^2^	Observed Power (1 − β)
Metacognitive prompts					
Time	0.88	4.83	0.035	0.12	0.57
Time × Ability group	0.99	0.323	0.574	0.01	0.09
Cognitive prompts					
Time	0.87	5.18	0.029	0.13	0.60
Time × Ability group	1.00	0.15	0.903	<0.001	0.05
Modelling					
Time	0.95	1.79	0.191	0.06	0.25
Time × Ability group	0.97	1.09	0.304	0.03	0.17

## Appendix A

**Table A1:** Schematic overview of the graduated prompts training procedure.

	Type of Prompt	Content of Prompt
1	Metacognitive	Activating task-related prior knowledge + check correct answer
2	Metacognitive	Activating prior knowledge regarding problem-solving strategy + check correct answer
3	Cognitive/task specific	Seeing similarities and differences A, B, C + check correct answer
4	Cognitive/task specific	Finding the relationship between A and B + check correct answer
5	Cognitive/task specific	Finding the relationship between A and C + check correct answer
6	Cognitive/modelling	Step-by-step modelling of correct solution

## Appendix B

**Table A2:** Psychometric characteristics of the pre-test and post-test in terms of the number of elements and transformations per item number, the corresponding *p*-values and the item-total correlations.

Item	Number of Elements	Number of Transformations	*p*-Values			Item-Total Correlations		
			Pre-Test	Post-Test Dynamic Testing	Post-Test Control	Pre-Test	Post-Test Dynamic Testing	Post-Test Control
1	2	3	0.27	0.53	0.46	0.52	0.63	0.78
2	2	3	0.28	0.68	0.46	0.77	0.84	0.78
3	2	3	0.28	0.71	0.43	0.64	0.94	0.74
4	2	3	0.12	0.71	0.54	0.55	0.94	0.78
5	2	4	0.14	0.59	0.34	0.56	0.75	0.74
6	2	4	0.33	0.53	0.46	0.71	0.71	0.72
7	2	4	0.21	0.47	0.34	0.59	0.63	0.73
8	2	4	0.21	0.56	0.29	0.71	0.77	0.69
9	3	6	0.69	0.44	0.34	0.40	0.61	0.77
10	3	6	0.21	0.65	0.54	0.64	0.86	0.88
11	3	6	0.27	0.50	0.26	0.58	0.72	0.69
12	3	6	0.07	0.59	0.37	0.52	0.79	0.61
13	3	6	0.15	0.35	0.26	0.52	0.53	0.68
14	3	6	0.21	0.44	0.23	0.62	0.68	0.57
15	3	6	0.31	0.38	0.23	0.73	0.48	0.52
16	4	6	0.15	0.50	0.34	0.62	0.66	0.70
17	4	6	0.35	0.56	0.23	0.70	0.55	0.55
18	4	7	0.19	0.41	0.20	0.67	0.63	0.52
19	4	7	0.06	0.64	0.34	0.43	0.80	0.70
20	5	8	0.08	0.25	0.11	0.55	0.45	0.55

**Table A3:** Descriptive statistics for age, Raven accuracy, pre-test accuracy, and post-test accuracy divided by ability group and condition.

		Dynamic Testing						Control					
		*M*	*SD*	min	max	Skew	Kurtosis	*M*	*SD*	min	max	Skew	Kurtosis
Age	Gifted	7.21	(0.43)	7	8	1.40	−0.06	7.18	(0.53)	6	8	0.26	0.74
	Average ability	7.23	(0.43)	7	8	1.57	0.50	7.33	(0.48)	7	8	0.76	−1.58
Raven accuracy	Gifted	40.71	(4.10)	32	48	−0.22	0.61	42.06	(3.92)	37	50	0.60	−0.40
	Average ability	28.95	(8.82)	10	40	−0.77	−0.34	29.71	(7.28)	15	41	−0.46	−0.42
Pre-test accuracy	Gifted	4.64	(4.41)	0	11	0.12	−1.95	5.59	(6.07)	0	20	0.94	0.18
	Average ability	3.18	(4.96)	0	18	1.67	2.43	2.62	(4.50)	0	16	1.92	3.28
Post-test accuracy	Gifted	13.36	(6.42)	0	19	−1.45	1.06	8.65	(6.30)	0	19	−0.05	−1.16
	Average ability	8.32	(7.30)	0	17	0.21	−1.91	5.05	(6.85)	0	20	0.81	−0.94
